# A multi-center randomized prospective study on the treatment of infant bronchiolitis with interferon α1b nebulization

**DOI:** 10.1371/journal.pone.0228391

**Published:** 2020-02-21

**Authors:** Lina Chen, Mingfang Shi, Quanmin Deng, Wenjun Liu, Qin Li, Piao Ye, Xiahui Yu, Benjin Zhang, Yuxia Xu, Xiaolan Li, Yao Yang, Min Li, Yi Yan, Zhe Xu, Jing Yu, Long Xiang, Xiaojun Tang, Guangping Wan, Qiang Cai, Li Wang, Bo Hu, Liang Xie, Gen Li, Lunyan Xie, Xiaoyun Liu, Chunyan Liu, Li Li, Lijie Chen, Xiaobin Jiang, Yana Huang, Si Wang, Jiang Guo, Yan Shi, Li Li, Xiaofang Wang, Zhiyong Zhao, Yan Li, Yanru Liu, Qiang Fu, Yan Zeng, Yan Zou, Dingyuan Liu, Deyun Wan, Tao Ai, Hanmin Liu

**Affiliations:** 1 Department of Pediatric Pulmonology and Immunology, West China Second University Hospital, Sichuan University, Chengdu, China; 2 Key Laboratory of Birth Defects and Related Diseases of Women and Children (Sichuan University), Ministry of Education, West China Second University Hospital, Sichuan University, Chengdu, China; 3 The First People’s Hospital of Yibin City, Yibin, China; 4 People’s Hospital of Deyang City, Deyang, China; 5 The Affiliated Hospital of Southwest Medical University, Luzhou, China; 6 Suining Central Hospital, Suining, China; 7 Sichuan Provincial Hospital for Women and Children, Chengdu, China; 8 Chongzhou City People’s Hospital, Chongzhou, China; 9 Dazhou Central Hospital, Dazhou, China; 10 Affiliated Hospital of North Sichuan Medical College, Nanchong, China; 11 Panzhihua Central Hospital, Panzhihua, China; 12 Liangshan First People’s Hospital, Xichang, China; 13 Sichuan Academy of Medical Sciences & Sichuan Provincial People’s Hospital, Chengdu, China; 14 The First People’s Hospital of Neijiang, Neijiang, China; 15 Guangyuan Central Hospital, Guangyuan, China; 16 Mianyang Central Hospital, Mianyang, China; 17 Chengdu First People’s Hospital, Chengdu, China; 18 Jianyang City People’s Hospital, Jianyang, China; 19 Meishan City People’s Hospital, Meishan, China; 20 The Second People’s Hospital of Yibin, Yibin, China; 21 The People’s Hospital of Leshan, Leshan, China; 22 Chengdu Fifth People’s Hospital, Chengdu, China; 23 Chengdu Women’s & Children’s Central Hospital, Chengdu, China; 24 Sinosource Biopharmaceutical Inc., Chengdu, China; Louisiana State University System, UNITED STATES

## Abstract

**Background:**

The respiratory syncytial virus (RSV) is the main cause of bronchiolitis in infants and interferon (IFN) α is a commercial antiviral drug. The nebulization of IFN α1b could be a viable treatment method. In this study, the therapeutic effects and safety of IFN α1b delivery via nebulization in infant bronchiolitis were investigated in this multi-center prospective study.

**Methods and findings:**

Bronchiolitis patients admitted to 22 hospitals who met the inclusion criteria were enrolled and randomly allocated to four groups: control, IFN Intramuscular Injection, IFN Nebulization 1 (1 μg/kg), and IFN Nebulization 2 (2 μg/kg) groups. All patients were observed for 7 days. The therapeutic effects and safety of different IFN delivery doses and delivery modes were evaluated. Coughing severity change, as scored by the researchers and parents, between days 1 and 3 was significantly different between the IFN Nebulization 2 and control groups. Lowell wheezing score change between days 3 and 5 was significantly different between IFN Nebulization 1 and control groups. There were no significant differences among the four groups regarding the number of consecutive days with fever, three-concave sign, fatigue and sleepiness, and loss of appetite. There were no cases of severe complications, no recurrence of fever, and no regression of mental status.

**Conclusions:**

IFN-α1b could more effectively alleviate coughing and wheezing in bronchiolitis. IFN-α1b nebulization had significant advantages in shortening the duration of wheezing and alleviating coughing.

## Introduction

Bronchiolitis is one of the most common acute lower respiratory tract inflammation diseases found among infants and young children, particularly during winter and spring [1]. In developing countries, there are approximately 34 million cases of bronchiolitis caused by the respiratory syncytial virus (RSV) among children below 5 years of age [2]. In the USA, bronchiolitis is the leading cause of hospitalization among infants younger than 1 year. The primary symptoms of bronchiolitis are wheezing and breathing difficulties, and severe cases can be life-threatening. More importantly, the results from several retrospective and prospective epidemiological studies have shown that the risk for childhood-onset asthma and allergic rhinitis is 5–6 times greater among patients who contracted RSV bronchiolitis in early childhood, significantly increasing the economic and psychological burden on society and the affected families [3].

There is currently a dearth of effective measures for the treatment of bronchiolitis. According to a summary by the "Expert Consensus on the Diagnosis, Treatment and Prevention of Bronchiolitis" [4] on therapeutic research in recent years, available drugs for etiological treatment with confirmed therapeutic efficacy are lacking, probably because bronchiolitis is mostly caused by the RSV, rhinovirus, metapneumovirus, and other respiratory viruses [5]. Furthermore, symptomatic treatments including glucocorticoids, bronchodilators, and hypertonic saline spray are lacking in high-level evidence-based support [6]. Although most cases are self-limited, the occurrence of severe cases and the long-term effects of potential subsequent asthma highlight the need for new treatment strategies.

In a review on the pathogenesis of bronchiolitis, our attention was drawn to the effects of interferon α (IFN- α) on the body’s activation of the immune system against viral infections. The invasion of exogenous viruses activates IFN-α expression via interaction with pattern recognition receptors. IFN-α is mainly produced in leukocytes, fibroblasts, and virus-infected tissue cells during the early stages of viral infection. By inducing the expression of various proteins with anti-viral and immune roles, IFN-α exerts anti-viral effects by limiting the spread of the virus at the affected area and suppressing viral replication [7]. Exogenous IFN-α is a marketed drug, but has limited clinical application in pediatrics because the drug is administered via intramuscular injection. However, recent advances in nebulization could provide an alternative method for drug delivery. Several preliminary studies have shown that IFN-α receptors are expressed in epithelial cells of the respiratory tract [8], and have found that nebulization of IFN-α1b (a major anti-viral subtype in the Chinese population) [9] led to significant symptom relief in animal models and bronchiolitis cases [10–11]. Therefore, we conducted a multi-center, randomized, open-label, prospective trial to investigate the therapeutic efficacy and safety of IFN-α1b nebulization in cases of infant bronchiolitis.

## Materials and methods

### Subjects and grouping

A randomized, open-label, controlled, multi-center trial was performed. The study was reviewed and approved by the Ethics Committee of West China Second University Hospital (No.2014-022). This clinical trial was registered in the Chinese Clinical Trial Registry (No.ChiCTR-IPR-14005413). The researchers who participated in the study had received extensive standardized training. Between December 2014 and April 2016, 22 tertiary general hospitals or specialized hospitals in Sichuan province enrolled 675 patients admitted for bronchiolitis that met the inclusion criteria. Informed consent was provided in writing by the patient’s parents. A researcher who was not involved in recruitment, diagnosis, and treatment evaluation informed the clinical staff of the patient’s grouping information based on the unified codes that were randomly assigned by a random number table according to the order of admission. The subjects were randomly divided into four groups, i.e., the control group, interferon intramuscular injection group, interferon low-dose nebulization group, and interferon high-dose nebulization group (the four groups will be referred to as the control group, IFN Injection, IFN Nebulization 1, and IFN Nebulization 2). All authors had access to information that could identify individual participants during or after data collection and pledged not to disclose this information.

### Inclusion, exclusion, and withdrawal criteria

#### Inclusion criteria

(1) Diagnosis of bronchiolitis at first hospital visit. Diagnostic criteria were based on the viral bronchiolitis diagnostic criteria and key hospitalization indications in the “Guidelines for management of community acquired pneumonia in children(the revised edition of 2013)” [12], and the “Expert Consensus of the Diagnosis, Treatment and Prevention of Bronchiolitis (2014 edition)” by The Subspecialty Group of Respiratory Diseases, The Society of Pediatrics, Chinese Medical Association [13]. (2) 0–12 months of age, and disease duration of ≤ 48 hours.

The exclusion criteria were as follows: (1) patients with severe bronchiolitis, including those who presented with shock or disorders of consciousness, recurrent apnea, or slow and irregular breathing; (2) patients using systemic glucocorticoids, anti-viral medications, traditional Chinese medicine (TCM) preparations with anti-viral effects, and other immunomodulators within 2 weeks before admission; (3) patients with bronchopulmonary dysplasia, shock, congenital heart disease, heart failure, liver, kidney and hematopoietic disorders, epilepsy and other central nervous system disorders; (4) patients with known allergies to interferon products.

The withdrawal criteria were as follows: (1) patients who did not meet the diagnosis for bronchiolitis after being included for treatment; (2) patients who presented with allergic reactions, adverse events, or serious adverse events during the trial; (3) patients who voluntarily requested to withdraw from the trial; (4) patients who were advised to discontinue the trial by their physician; (5) patients with serious protocol violations.

### Trial protocol

The four groups were administered strictly limited symptomatic treatment, which included antipyretics, analgesics, sputum aspiration if necessary during oxygen inhalation, nebulized inhalation of budesonide with β2 receptor agonists, and orally administered β2 receptor agonists. Any use of non-study anti-viral agents was strictly prohibited, and detailed records were made when systemic glucocorticoids were used. The control group was only administered symptomatic treatment. In addition to the symptomatic treatments, patients in IFN Injection were administered an intramuscular injection of IFN-α1b (from Kexing Biotech Co., Ltd, Shenzhen, China, 10 μg, once daily), patients in IFN Nebulization 1 were administered IFN-α1b (1 μg/kg, dose chosen and revised from [11]) dissolved in 4 mL of 0.9% saline for injection, compressed and nebulized (by nebulizers from PARI GmbH, Starnberg, Germany), for 20 min twice daily, and patients in IFN Nebulization 2 were administered nebulized IFN-α1b (2 μg/kg, dose chosen and revised from [11]) for 20 min twice daily. The duration of IFN-α1b use for all three treated groups was 7 days. The protocol can be accessed by contacting the corresponding authors.

### Outcome measures

1. General condition
  The patients’ body temperature, respiratory rate, pulse, and other vital signs were recorded daily at regular interval during the observation period.
2. Changes in coughing, wheezing, oxygenation, rales, and the three-concave sign (depressions in the suprasternal fossa, supraclavicular fossa, and intercostal space) were observed and recorded. Coughing was evaluated using a visual analogue scale, which was assessed by the same ward doctor and parents of the patient at fixed intervals. Wheezing was evaluated by the ward doctor using the Respiratory Distress Assessment Instrument (Lowell wheezing scores) as shown in Table 1. Oxygenation was evaluated via transcutaneous measurement of oxygen saturation. Other factors were evaluated daily by the ward doctor at fixed intervals.
3. The safety indicators included the patient’s temperature changes, incidence of flu-like symptoms, rash, fatigue and sleepiness, loss of appetite, and other adverse events during the treatment period.

**Table 1:** Respiratory Distress Assessment Instrument (Lowell wheezing score, From [14]).

Signs	Points					Maximum Points
	0	1	2	3	4	
**Expiration wheezing**	None	End	1/2	3/4	All	4
**Inspiration wheezing**	None	Part	All			2
**Location wheezing**	None	Segmental: 2 lung fields	Diffuse: 3 lung fields			2
**Supraclavicular retractions**	None	Mild	Moderate	Marked		3
**Intercostal retractions**	None	Mild	Moderate	Marked		3
**Subcostal retractions**	None	Mild	Moderate	Marked		3

The degree of symptom relief in each group was determined by measuring the differences between days 1 and 3, days 3 and 5, and days 5 and 7. Comparisons among the four groups or between two groups were used to evaluate the effect of different methods of IFN treatment on symptom relief by statistical methods.

### Statistical methods

SPSS 16.0 was employed to perform data processing. Measurement data were expressed using $\overset{-}{x}\pm sem$. The Kolmogorov–Smirnov test was used to test for normality of data distribution. For skewed data, comparison among the four groups was performed using the Kruskal–Wallis test and pairwise comparisons were performed using Mann–Whitney U test. For normally distributed data, comparison among the four groups was performed using one-way analysis of variance. Pairwise comparisons were performed using the least significant difference test. Count data were analyzed using the chi-suqared test. The cut-off value of *P* was 0.05 and was corrected by the Bonferroni method for multiple comparisons.

## Results

### General condition of included patients

**Fig 1:**
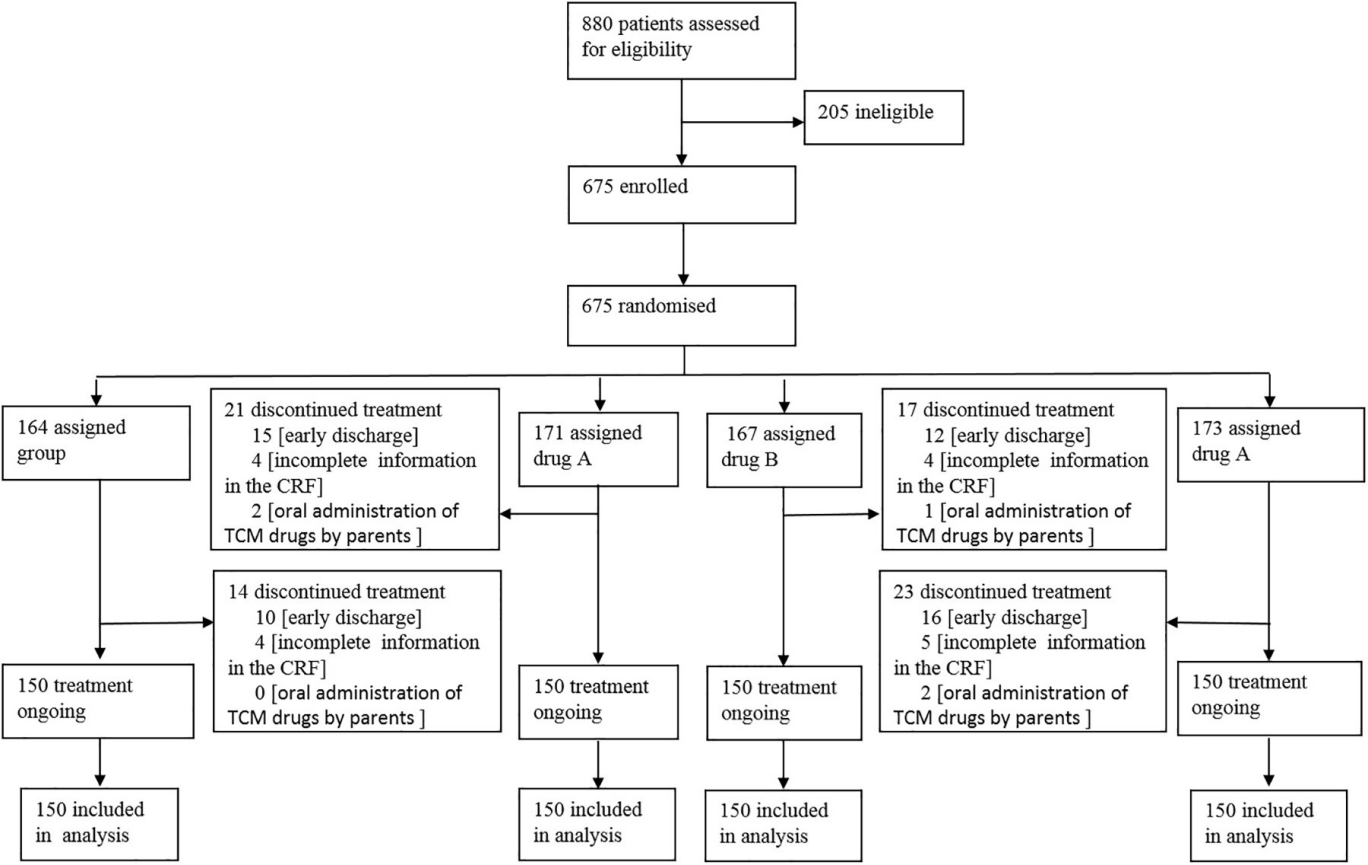
The flow diagram of this study.

A total of 675 patients were enrolled in 22 centers, including 600 valid and 75 dropout cases, for a dropout rate of 11.1%. The reasons for dropout included requests by parents for early discharge, incomplete information in the CRF (Case Report Form), and oral administration of TCM drugs by parents. The 600 valid cases were divided into four groups, with 150 cases in each group (Fig 1). The patients included 407 boys and 193 girls, with a male: female ratio of 2.1:1. The age range was 243.91±7.16 days, and 97.8% of the patients were of Han ethnicity. The duration of disease upon enrollment was 1.28±0.48 days.

The baseline data of all groups were analyzed. There were no significant differences in age, the coughing score as assessed by researchers, coughing score as assessed by parents, Lowell wheezing score, and oxygen saturation among the groups (Table 2). There were no significant differences in gender, fever, abnormal chest radiographic findings, and antibiotics using upon enrollment among the groups. The incidence of the three-concave sign in the control group was significantly lower than those in the other groups (Table 3).

**Table 2:** The comparison of baseline data by the Kruskal—Wallis test.

Parameters	IFN Injection	Control	IFN Nebulization 1	IFN Nebulization 2	χ^2^	*P*
**Age**	245.49±14.41	242.29±14.38	244.15±13.36	244.17±15.10	0.465	0.926
**Researchers’ coughing score**	6.15±0.156	5.79±0.153	5.90±0.152	5.98±0.148	2.867	0.413
**Parents’ coughing score**	6.88±0.157	6.33±0.169	6.45±0.155	6.58±0.157	4.507	0.212
**Lowell wheezing score**	4.99±0.197	4.82±0.170	5.30±0.175	4.75±0.188	8.671	0.034
**Oxygen saturation**	96.205±0.2256	95.945±0.2382	94.583±0.7211	96.164±0.2202	4.679	0.197

**Table 3:** The comparison of baseline data by the chi-squared test.

	Gender		Fever		Three-concave sign		Abnormal chest X-ray		Antibiotics usage in treatment	
	M	F	Yes	No	Yes	No	Yes	No	Yes	No
**IFN Injection**	97	53	44	106	40	110	127	23	39	111
**Control**	104	46	51	99	31	119	128	22	44	106
**IFN Nebulization 1**	111	39	59	91	57	93	140	10	37	113
**IFN Nebulization 2**	95	55	50	100	31	119	128	22	43	107
χ^2^	4.850		3.387		15.428		6.839		1.103	
*P*	0.183		0.336		0.001		0.077		0.776	

### Comparison of coughing relief

The comparison of coughing scores as assessed by the researchers between different time points indicated that the score differences between days 1 and 3, and between days 3 and 5 were statistically significant among the four groups, whereas differences between days 5 and 7 were not (Table 4, *p* values were 0.003, 0.005, and 0.280, respectively).

**Table 4:** The comparisons for the differences in coughing scores as assessed by the researchers among the four groups by the Kruskal—Wallis test.

Parameters	IFN Injection	Control	IFN Nebulization 1	IFN Nebulization 2	χ^2^	*P*
**The differences of researchers’ coughing scores of days 1 and 3**	1.88±0.16	1.32±0.10	1.58±0.13	2.07±0.14	14.105	0.003
**The differences of researchers’ coughing scores of days 3 and 5**	2.04±0.12	1.59±0.12	2.07±0.11	1.96±0.13	12.940	0.005
**The differences of researchers’ coughing scores of days 5 and 7**	1.87±0.14	1.48±0.10	1.47±0.13	1.38±0.20	3.834	0.280

Pairwise comparisons between pairs of the four groups indicated that with regard to the score difference between days 1 and 3, IFN Nebulization 2 significantly differed from the control group (*p* value was <0.001.), whereas IFN injection and IFN Nebulization 1 did not significantly differ from the control group. With regard to the score differences between days 3 and 5, IFN Injection, IFN Nebulization 1, and IFN Nebulization 2 were all significantly different than the control group (*p* = 0.004, 0.003, and 0.009, respectively) (Table 5).

**Table 5:** The P values of pairwise comparisons for the differences in coughing scores as assessed by the researchers by the Mann—Whitney U test. (Z values in the parenthesis).

Parameters	IFN Injection vs Control	IFN Injection vs IFN Nebulization 1	IFN Injection vs IFN Nebulization 2	Control vs IFN Nebulization 1	Control vs IFN Nebulization 2	IFN Nebulization 1 vs IFN Nebulization 2
**The differences of researchers’ coughing scores of days 1 and 3**	0.018(-2.359)	0.382(-0.873)	0.248(-1.155)	0.126(-1.529)	<0.001(-3.636)	0.028(-2.192)
**The differences of researchers’ coughing scores of days 3 and 5**	0.004(-2.844)	0.955(-0.057)	0.811(-0.239)	0.003(-2.951)	0.009(-2.601)	0.811(-0.239)

When comparing the coughing scores as assessed by the parents between different time points, the score difference between days 1 and 3 was statistically significant among the four groups, whereas the scores between days 3 and 5 and 5 and 7 were not significantly different (Table 6, *p* values were <0.001, 0.042, and 0.075, respectively).

**Table 6:** The comparisons for the differences in coughing scores as assessed by the parents among the four groups by Kruskal—Wallis test.

Parameters	IFN Injection	Control	IFN Nebulization 1	IFN Nebulization 2	^χ2^	*P*
The differences of parents’ coughing score of days 1 and 3	2.29±0.19	1.41±0.11	1.82±0.14	2.46±0.16	25.727	<0.001
The differences of parents’ coughing score of days 3 and 5	2.24±0.13	1.83±0.13	2.22±0.12	2.21±0.15	8.192	0.042
The differences of parents’ coughing score of days 5 and 7	1.95±0.15	1.45±0.11	1.50±0.12	1.54±0.21	6.904	0.075

Pairwise comparisons among the four groups indicated that with regard to the score differences between days 1 and 3, IFN Injection and IFN Nebulization 2 significantly differed from the control group (*p* values were 0.001 and <0.001, respectively). With regard to the score differences between days 3 and 5, there were no significant results when comparing three IFN groups to the control group, and there were no statistically significant differences in the pairwise comparisons among the three IFN groups (Table 7).

**Table 7:** The P values of pairwise comparisons for the differences in coughing score as assessed by the parents by Mann—Whitney U test. (Z values in the parenthesis).

Parameters	IFN Injection vs Control	IFN Injection vs IFN Nebulization 1	IFN Injection vs IFN Nebulization 2	Control vs IFN Nebulization 1	Control vs IFN Nebulization 2	IFN Nebulization 1 vs IFN Nebulization 2
**The differences of parents’ coughing score of days 1 and 3**	0.001(-3.326)	0.187(-1.320)	0.254(-1.140)	0.020(-2.325)	<0.001(-4.891)	0.005(-2.781)
**The differences of parents’ coughing score of days 3 and 5**	0.008(-2.636)	0.679(-0.414)	0.255(-1.138)	0.021(-2..314)	0.160(-1.405)	0.434(-0.783)

### Comparison of wheezing relief

Comparison of differences in Lowell wheezing scores between different time points among the four groups indicated that the score difference between days 3 and 5 was statistically significant, whereas the differences between days 1 and 3 and 5 and 7 were not (Table 8, *p* values were <0.001, 0.165, and 0.149, respectively).

**Table 8:** The comparisons for the differences in the Lowell wheezing scores among the four groups by the Kruskal—Wallis test.

Parameters	IFN Injection	Control	IFN Nebulization 1	IFN Nebulization 2	χ^2^	P
**The differences of Lowell wheezing scores of days 1 and 3**	1.60±0.17	1.38±0.14	1.64±0.15	1.91±0.16	5.098	0.165
**The differences of Lowell wheezing scores of days 3 and 5**	1.90±0.17	1.36±0.16	2.27±0.14	1.74±0.15	25.331	<0.001
**The differences of Lowell wheezing scores of days 5 and 7**	1.57±0.18	1.68±0.22	1.14±0.19	1.34±0.21	5.337	0.149

Pairwise comparisons among the four groups indicated that there were no significant differences with regard to the score differences between days 1 and 3. With regard to the score differences between days 3 and 5, only IFN Nebulization 1 significantly differed from the control group (Table 9, the *p* value was <0.001).

**Table 9:** The P values of pairwise comparisons for the differences in the Lowell wheezing scores by the Mann—Whitney U test. (Z values in the parenthesis).

Parameters	IFN Injection vs Control	IFN Injection vs IFN Nebulization 1	IFN Injection vs IFN Nebulization 2	Control vs IFN Nebulization 1	Control vs IFN Nebulization 2	IFN Nebulization 1 vs IFN Nebulization 2
**The differences of Lowell wheezing scores of days 1 and 3**	0.785(-0.273)	0.382(-0.874)	0.061(-1.875)	0.399(-0.843)	0.044(-2.015)	0.260(-1.127)
**The differences of Lowell wheezing scores of days 3 and 5**	0.007(-2.717)	0.065(-1.845)	0.513(-0.654)	<0.001(-4.803)	0.018(-2.374)	0.005(-2.839)

### Safety observations

There was no report of IFN administration related side effects, such as pain, swelling or redness in injection area, and bronchospasm. To facilitate statistical analysis, post-treatment rash, recurrence of fever after abatement, and regression of mental status were included as adverse events. The results of this analysis indicated no serious complications, no recurrence of fever after abatement, and no regression of mental status. Three patients presented with rash; one case appeared after 2 days in the control group, and two cases appeared between 4 and 6 days in IFN Nebulization 1. Two cases showed mild extents of poor mental status and poor appetite 5 days after treatment in IFN Nebulization 1, which were relieved after 1 day of symptomatic treatment.

## Discussion

### Feasibility of treatment with IFN nebulization

Evidence-based research indicates that there are limited drugs with confirmed clinical efficacy for bronchiolitis. IFN is one of the key endogenous anti-viral factors in the human body. Studies have shown that the anti-viral effects exerted by IFN-α1b are primarily achieved by inducing the production anti-viral proteins and activation of cell-mediated immunity. Li et al. reported the treatment of an RSV-infected mouse model with nebulized IFN-α1b [10], and their results indicated that IFN-α1b could significantly inhibit spreading of the virus, and mitigate interstitial inflammation of the lungs. Numerous small-sample clinical case studies have shown that IFN-α1b could effectively alleviate clinical manifestations. A multi-center study by Shang et al. also showed similar findings [11].

Numerous studies have demonstrated the therapeutic efficacy of IFN intramuscular injections for bronchiolitis [8]. As the lesion site of bronchiolitis is located in the bronchioles, theoretically, the local action of drugs delivered via nebulization could have the following advantages: (1) rapid onset of therapeutic effect due to the direct path of action; (2) direct drug action due to the rich variety of drug receptors in the respiratory mucosa and submucosa; (3) reduced use of systemic medication, with incidental airway humidification and sputum dilution; (4) reduced trauma compared to intramuscular injection in pediatric patients, and greater patient compliance. Therefore, the inhalation of nebulized IFN has potential prospects for future clinical application.

Drug delivery via nebulization must satisfy some basic requirements. The nebulized particles must have an appropriate size, the biological properties of the nebulized IFN particles must be stable, and the drug must be able to enter the mucosal epithelial cells to exert its effects. A pharmacokinetic study on the inhalation of nebulized IFN-α1b in rabbits indicated that compared to conventional intramuscular injection, inhalation of nebulized IFN-α1b significantly increased the concentration of IFN-α1b in the lungs, with longer pulmonary residence time. The concentration of IFN- α1b at 12 h was four times higher than that in intramuscular injection, the time of peak blood concentration was significantly delayed to 8 h, and the drug levels in renal tissue consistently remained at low concentrations. This indirectly demonstrates the clinical feasibility of IFN-α1b delivery via nebulization [15].

### Efficacy analysis of bronchiolitis treatment with IFN-α1b nebulization

In this study, we optimized the protocol design to reduce data biases. We strictly controlled the random allocation of cases by ensuring the mutual independence between the personnel involved in clinical data collection and those involved in group allocation. Further, we reduced the potential bias caused by differences in the natural disease duration of bronchiolitis by limiting the disease duration at enrollment to within 48 h. Furthermore, by selecting infants younger than 1 year old as the enrollment age ensured that all patients were approximately 8 months old upon enrollment. We also reduced subjectivity in the observation of clinical symptoms by asking the patient’s parents and physicians to provide independent scores on the two primary symptoms, coughing and wheezing, which were evaluated using the internationally-accepted visual analogue scale and Lowell scale respectively. Secondary symptoms were evaluated based on the number of consecutive days of occurrence. Finally, strict restrictions were imposed on the treatment measures.

Based on the above-mentioned strategies, our findings indicate that in terms of the baseline data upon enrollment, this study employed randomized, prospective enrollment, which excluded patients with severe bronchiolitis and comorbidities based on the pre-set exclusion criteria.

It should be noted that the results at enrollment indicate significant differences in the three-concave sign. Specifically, the control group had the lowest incidence of three-concave sign. These differences, however, do not affect the interpretation of our findings.

The scores provided by the researchers and patients’ parents for the primary symptom, coughing, showed overall consistent results. Both results indicate that high level IFN nebulization administered within 2 days of symptom onset could more significantly alleviate coughing compared to the control treatment. Moreover, between-group comparisons showed that on days 3 and 5 of treatment, the alleviation of coughing was similar between the intramuscular injection and nebulization groups.

With regard to the primary symptom, wheezing, the Lowell scores indicates that there were no significant differences in the score differences between days 1 and 3. On day 5 of treatment, the efficacy of low IFN nebulization was superior to that of the control treatment, but there were no significant differences among the treated groups with regard to delivery mode.

The results of our prospective cohort study showed that compared to the control group, administration of IFN-α1b could effectively alleviate wheezing and coughing in bronchiolitis. With regard to the drug delivery method, high-dose IFN-α1b nebulization showed significant advantages in shortening the duration of wheezing and alleviating coughing, while also avoiding the discomfort and inconvenience of the patients and their parents caused by intramuscular injections. We did not encounter any patient safety issues pertaining to the use of IFN-α1b nebulization during the study.

There are several limitations in this study. First, the patients were hospitalized for 7 days. It is common in China to ensure that there is no symptom recurrence. In this study, there was no further apparent improvement in the scores after the5th day. Second, the study was multi-center, including numerous hospitals located in developing areas. The recruitment criteria could not be strictly followed due to lack of expertise of some clinical doctors, which will be improved by better training in future studies. Third, the test of virus was not analyzed in this study as data was unavailable in some centers. It is very important to learn viral etiology for bronchiolitis treatment. Meanwhile, the data of the need of supportive care was lack in this study. It is important to assess the efficacy of IFN-α1b. These data will be collected in future studies.

In the future, we will aim to observe the immunological changes in the patients, and follow-up on the recurrence of wheezing. This will provide a more comprehensive clinical basis for the treatment of bronchiolitis with IFN-α1b nebulization in pediatric patients.

## Supporting information

S1 Table CONSORT 2010 checklist.doc. (DOCX)
