# Correction: A multi-center randomized prospective study on the treatment of infant bronchiolitis with interferon α1b nebulization

**DOI:** 10.1371/journal.pone.0231911

**Published:** 2020-04-10

**Authors:** Lina Chen, Mingfang Shi, Quanmin Deng, Wenjun Liu, Qin Li, Piao Ye, Xiahui Yu, Benjin Zhang, Yuxia Xu, Xiaolan Li, Yao Yang, Min Li, Yi Yan, Zhe Xu, Jing Yu, Long Xiang, Xiaojun Tang, Guangping Wan, Qiang Cai, Li Wang, Bo Hu, Liang Xie, Gen Li, Lunyan Xie, Xiaoyun Liu, Chunyan Liu, Li Li, Lijie Chen, Xiaobin Jiang, Yana Huang, Si Wang, Jiang Guo, Yan Shi, Li Li, Xiaofang Wang, Zhiyong Zhao, Yan Li, Yanru Liu, Qiang Fu, Yan Zeng, Yan Zou, Dingyuan Liu, Deyun Wan, Tao Ai, Hanmin Liu

The affiliation for the forty-fourth author is incorrect. Tao Ai is not affiliated with #23 but with #24: Chengdu Women’s & Children’s Central Hospital, Chengdu, China.

Additionally, Tao Ai should be noted as having also contributed equally to this work with: Lina Chen, Mingfang Shi, Quanmin Deng, Wenjun Liu, Qin Li, Piao Ye, Xiahui Yu, Benjin Zhang, Yuxia Xu, Xiaolan Li, Yao Yang, Min Li, Yi Yan, Zhe Xu, Jing Yu, Long Xiang, Xiaojun Tang, Guangping Wan, Qiang Cai, Li Wang, Bo Hu, Tao Ai.
